# Cognitive Reserve in Early Onset Alzheimer's Disease

**DOI:** 10.1002/alz70857_106494

**Published:** 2025-12-26

**Authors:** Yeshin Kim, Hyemin Jang, Jae‐Won Jang, So Young Moon, Eun‐Joo Kim, Helmet T. Karim, Sang Joon Son

**Affiliations:** ^1^ College of Medicine, Kangwon National University, Chuncheon, Gangwon‐do, Korea, Republic of (South); ^2^ Seoul National University Hospital, Seoul National University College of Medicine, Jongno‐gu, Seoul, Korea, Republic of (South); ^3^ Ajou University School of Medicine, Suwon, Gyeonggi‐do, Korea, Republic of (South); ^4^ Pusan Medical University Hospiral, Pusan, Korea, Republic of (South); ^5^ University of Pittsburgh, Pittsburgh, PA, USA; ^6^ Ajou University School of Medicine, Suwon, Gyeonggido, Korea, Republic of (South)

## Abstract

**Background:**

Cognitive Reserve (CR) is a concept explaining the discrepancy between the extent of brain pathology and its clinical manifestations. Understanding CR and its associated factors is important, as increasing CR can be applied to dementia prevention strategies. While related factors of CR have been previously studied, they have been rarely explored in Early Onset Alzheimer's Disease (EOAD), a form of Alzheimer's that manifests at an earlier age and progresses rapidly. This study aims to measure CR using brain age residuals and identify factors related to CR in EOAD patients

**Method:**

Data were collected from the Longitudinal study of Early Onset Dementia And Family members (LEAF) cohort, encompassing 179 EOAD patients. CR at the time of enrollment was quantified as the brain age gap (BAG). BAG was derived from a machine‐learning‐based brain age model, defined as the difference between predicted brain age and chronological age, using previously suggested methods with MRI T1 images. We then examined the effect of lifelong exposure factors on CR, such as education, job experiences, leisure activities, and psychiatric factors like depression and stress. Multiple linear regression models were used to identify the association between these factors and BAG, adjusting for age and sex.

**Result:**

Average ages of participants were 61.5 years, and 69.2% were female. Mean brain age were 78.0 years and mean BAG was ‐20.0 years. BAG showed significant associations with lifetime leisure activities (*p* = 0.011). Higher levels of leisure activities were positively correlated with greater BAG, while other factors did not show a significant association with BAG.

**Conclusion:**

This study highlights the potential modifiable factors influencing CR in EOAD patients, suggesting that enhancing leisure activities could improve CR in EOAD patients. These findings can inform strategies for dementia prevention and provide a foundation for further research into CR in EOAD patients.

